# Study of the Physical–Chemical, Thermal, Structural, and Rheological Properties of Four High Andean Varieties of Germinated *Chenopodium quinoa*

**DOI:** 10.3390/polym17030312

**Published:** 2025-01-24

**Authors:** Betsy S. Ramos-Pacheco, Carlos A. Ligarda-Samanez, David Choque-Quispe, Yudith Choque-Quispe, Aydeé M. Solano-Reynoso, Katia Choque-Quispe, Henry Palomino-Rincón, Fredy Taipe-Pardo, Diego E. Peralta-Guevara, Elibet Moscoso-Moscoso, Yasmine Diaz-Barrera, Henrry Wilfredo Agreda-Cerna

**Affiliations:** 1 Department of Agroindustrial Engineering, Universidad Nacional José María Arguedas, Andahuaylas 03701, Peru; caligarda@unajma.edu.pe (C.A.L.-S.); dchoque@unajma.edu.pe (D.C.-Q.); hpalomino@unajma.edu.pe (H.P.-R.); ftaipe@unajma.edu.pe (F.T.-P.); emoscoso@unajma.edu.pe (E.M.-M.); ydiaz@unajma.edu.pe (Y.D.-B.); 2 Food Nanotechnology Research Laboratory, Universidad Nacional José María Arguedas, Andahuaylas 03701, Peru; 3 Water and Food Treatment Materials Research Laboratory, Universidad Nacional José María Arguedas, Andahuaylas 03701, Peru; 4 Department of Environmental Engineering, Universidad Nacional José María Arguedas, Andahuaylas 03701, Peru; ychoque@unajma.edu.pe; 5 Department of Basic Sciences, Universidad Nacional José María Arguedas, Andahuaylas 03701, Peru; amsolano@unajma.edu.pe; 6 Department of Accounting and Finance, Universidad Nacional José María Arguedas, Andahuaylas 03701, Peru; kchoque@unajma.edu.pe; 7 Agroindustrial Research Laboratory, Universidad Nacional José María Arguedas, Andahuaylas 03701, Peru; 8 Department of Business Sciences, Universidad Nacional José María Arguedas, Andahuaylas 03701, Peru; hagreda@unajma.edu.pe

**Keywords:** *Chenopodium quinoa*, germination, bioavailability, biopolymer

## Abstract

*Chenopodium quinoa*, a high Andean grain with excellent nutritional value and complex molecular structure, presents significant challenges in the bioavailability of nutrients and the functionality of its components. Germination as a biotechnological strategy generated significant modifications in four varieties of quinoa. The ungerminated and germinated samples’ physical–chemical, thermal, structural, and rheological properties were determined. Results showed increases in protein bioavailability (14.13% in Black Collana Quinoa (BCQ) and 12.79% in Red Pasankalla Quinoa (RPQ)), phenolic compounds (30.81 mg Gallic Acid Equivalent/100 g in RPQ), flavonoids (108.53 mg Quercetin Equivalent/100 g in Yellow Marangani Quinoa (YMQ)), and antioxidant capacity (up to 241.43 μmol Trolox Equivalent/g in BCQ). Thermal analysis showed increases in gelatinization temperature (57.53 °C to 59.45 °C in RPQ) and a reduction in enthalpy (1.38 J/g to 0.67 J/g). Structural analysis showed similar functional groups, but variation in spectra intensity was related to starches and proteins. Rheological properties exhibited pseudoplastic behavior at 80 °C. Principal component analysis showed a clear difference between germinated and non-germinated samples. The germination process significantly modified quinoa, improving its nutritional and functional properties and generating new opportunities for its application in the development of biodegradable materials and functional foods.

## 1. Introduction

The growing global demand for functional foods and sustainable materials has brought interest to Andean crops such as *Chenopodium quinoa*, a high-Andean pseudocereal that has gained worldwide recognition due to its excellent nutritional and functional value. As a crop originating in the Andean regions of South America, high-Andean quinoa varieties have demonstrated great adaptability and resilience to adverse environmental conditions, making them a valuable resource [1,2]. Quinoa is rich in protein and contains all essential amino acids, dietary fiber, bioactive compounds, vitamins, and minerals [3,4]. In addition to these characteristics, quinoa presents thermal and structural properties that make it a potential resource for developing highly functional biodegradable biomaterials [5,6,7].

Despite its many advantages, quinoa faces challenges, such as the low bioavailability of certain nutrients, the presence of antinutrients, and limitations in the functionality of its biopolymers. In this context, germination emerges as a promising biotechnological strategy, as it induces modifications that improve the nutritional and functional properties of the grains [8,9,10,11,12,13,14].

Recent studies have reported increases in antioxidant bioactivity, improved bioavailability of proteins and minerals, and modifications in the structure of germinated grains. These findings highlight the potential of quinoa for applications in functional foods and advanced materials, including biodegradable structures, controlled release systems, and biomaterials with innovative applications in smart packaging and sustainable nanotechnology [15,16,17,18,19,20]. The studies’ results provide a scientific basis for the development of high-value products with improved functionality, helping to meet current industry demands in terms of sustainability and technical performance.

This study focused on analyzing the physical–chemical, thermal, structural, and rheological properties of four Andean varieties of germinated quinoa and evaluating the changes in the properties. The results obtained provide key scientific information to develop biodegradable and functional materials and address sustainability challenges in the industry.

## 2. Materials and Methods

### 2.1. Materials

The quinoa varieties selected for this study were Yellow Marangani (YMQ), Black Collana (BCQ), Red Pasankalla (RPQ), and Lord of Orchard (LOQ). The varieties were selected based on their agricultural relevance in the Andean region and represent a range of colors, compositions, and grain structures.

The chemicals used were sodium carbonate (Spectrum, NB, Canada), gallic acid (Merck, Darmstadt, Germany), Folin–Ciocalteau reagent (Himedia, Dindori, India), methanol (JT Baker, Mexico City, Mexico), quercetin (Sigma Aldrich, St. Louis, MO, USA), aluminum chloride (Sigma Aldrich, St. Louis, MO, USA), Trolox (Sigma Aldrich, St. Louis, MO, USA), DPPH reagent (HiMedia, Mumbai, India), and potassium bromide IR grade (Thermo Fisher Scientific, Garfield, NJ, USA).

### 2.2. Germination

**Figure 1:**
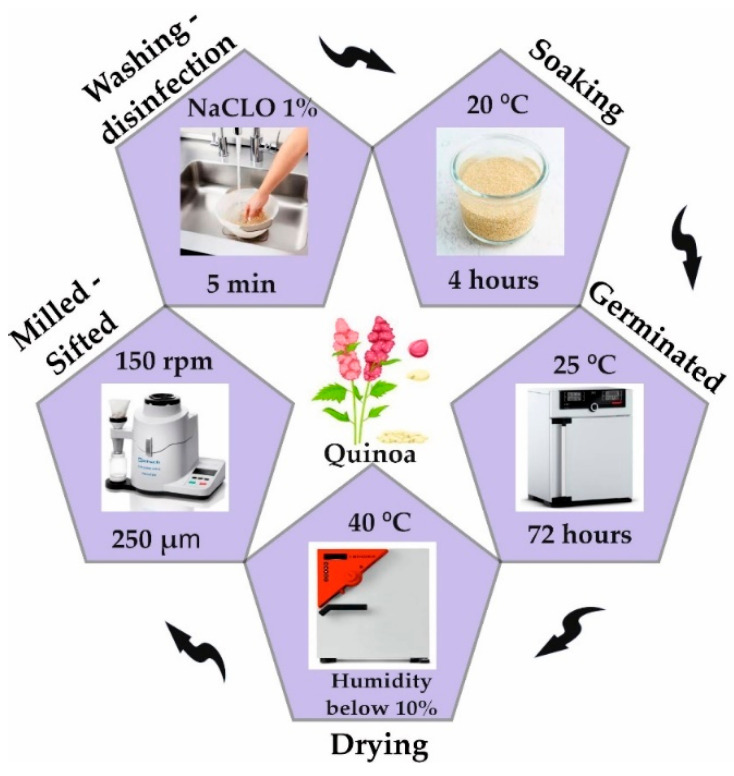
Quinoa germination process.

The grains (Figure 1) were washed and disinfected in a 1% sodium hypochlorite solution for 5 min. Subsequently, they were soaked in distilled water for 12 h, reaching a moisture content close to 40%. Then, the hydrated grains were placed in a FOC 200 E humid chamber (Velp Scientifica TM, Usmate Velate, Italy) for 72 h at 28 °C to promote their germination. Once this stage was completed, the germinated grains were dried in a FED 115 forced-convection oven (BINDER, Tuttlingen, Germany) at 40 °C until a less than 10% moisture content was achieved. Finally, they were ground using a Twister cyclone mill (Retsch, Haan, Germany) at 150 rpm for 3 min and sieved with a 250 µm mesh [21].

### 2.3. Physical–Chemical Properties

#### 2.3.1. Color

The color was determined using a Konica Minolta CR5 colorimeter, evaluating lightness (L*), chroma a* and b*. In addition, the whiteness index (WI) was calculated following Equation (1):

(1) $$WI=100-\sqrt{{\left(100-L*\right)}^{2}+{a*}^{2}+{b*}^{2}},$$

#### 2.3.2. Water Activity

Water activity was measured with a portable HygroPalm23-AW (Rotronic, Bassersdorf, Switzerland). The device was calibrated. Afterward, 5 g of the sample was taken and placed in a disposable container. The probe was inserted, and the reading was taken [22].

#### 2.3.3. Particle Size

Particle size and distribution were evaluated by laser light scattering using a Mastersizer 3000 (Malvern Instruments, Worcestershire, UK). The sample was dispersed in isopropanol, and the mean particle size was expressed as the volume median diameter (D4;3) [23]. The polydispersity was determined through the amplitude index (Span), calculated with Equation (2) [12,24]:

(2) $$Span=\frac{D\left(0.9\right)-D(0.1)}{D(0.5)}$$

where $D\left(0.1\right),\;D\left(0.5\right),$ and $D\left(0.9\right)$ correspond to the relative diameters at 10%, 50%, and 90% of the accumulated size distribution.

#### 2.3.4. Total Phenolics and Flavonoids

The total content of phenolic compounds was estimated using the Folin–Ciocalteu reagent. In total, 0.9 mL of the extract was mixed with 2.4 mL of deionized water, 0.15 mL of 20% sodium carbonate, and 0.3 mL of 0.25 N Folin–Ciocalteu reagent. Samples were analyzed at a wavelength of 755 nm using a spectrophotometer (Genesys 150, Thermo Fisher Scientific, Waltham, MA, USA). Gallic acid (GA) was used as a reference standard [23].

The total flavonoid content was determined using a standard solution of quercetin in ethanol. In total, 90 µL of the extract was mixed with 4.81 mL of methanol and 100 µL of aluminum chloride. The samples were read at 450 nm in a spectrophotometer (Genesys 150, Thermo Fisher Scientific, Waltham, MA, USA). The results were expressed as mg of quercetin equivalent/100 g of sample [25].

#### 2.3.5. Antioxidant Capacity by DPPH

The antioxidant activity assay by DPPH was performed using the stable radical 2,2-diphenyl-1-picrylhydrazyl. In total, 150 µL of the extract was mixed with 2850 µL of a diluted DPPH solution, and readings were taken at a wavelength of 515 nm in a spectrophotometer (Genesys 150, Thermo Fisher Scientific, Waltham, MA, USA). Trolox was used as a reference standard [23].

#### 2.3.6. Proximate Chemical Composition

The proximate chemical composition of the samples was determined following the methods established by the Association of Official Analytical Chemists (AOAC). The analyses included crude protein (AOAC 955.04), ash (AOAC 942.05), moisture (AOAC 925.10), and crude fat (AOAC 2003.05). The total carbohydrate content was calculated by difference.

#### 2.3.7. Mineral Micronutrients

Mineral micronutrient analysis was performed using an atomic absorption spectrometer model A6800 (Shimadzu, Kyoto, Japan). In total, 5 g of sample was incinerated for 5 h at 550 °C to obtain ash. Then, 0.1 g of the sample was digested in a microwave (SCP Science, Miniwave, Montreal, QC, Canada) using nitric acid. The mineral elements were identified by their characteristic emission spectra, compared to a standard curve [26].

### 2.4. Thermal Properties

#### 2.4.1. Temperature and Enthalpy of Gelatinization

Thermal properties were evaluated using a differential scanning calorimeter (DSC). In total, 2.0 mg of the sample was weighed into an aluminum pan, and 10 µL of distilled water was added. The recipient was sealed tightly and equilibrated at room temperature for 1 h. Subsequently, heating was programmed at a rate of 5 °C/min in a range of 40 to 100 °C, using an empty sealed recipient as a reference. The peak gelatinization temperature (Tp) and transition enthalpy (ΔH) were automatically calculated [18].

#### 2.4.2. Thermal Stability

Thermal stability was analyzed by thermogravimetry, using a thermobalance connected to a standard furnace with a nitrogen flow rate of 50 mL/min. The thermogram was generated by recording the sample’s weight loss and temperature at four-second intervals (Manals-Cutiño et al., 2011) [27].

### 2.5. Structural Properties

#### Functional Groups

The analysis was performed by mixing the previously dried sample with dehydrated KBr in a mortar and then pressing it to form a thin film. The sample’s IR spectrum was obtained using a Nicolet IS50 FTIR spectrophotometer (ThermoFisher, Waltham, MA, USA), using the transmission module, in a wavenumber range between 4000 and 400 cm^−1^ [10,26].

### 2.6. Rheology Properties

Rheological properties were determined using an Anton Paar rotational rheometer, model MCR702e (Graz, Austria), with a concentric cylinder geometry. Measurements were performed at a controlled shear rate of 1 to 200 s⁻^1^, evaluated at temperatures of 40, 60, and 80 °C, using a 5% sample suspension. The data obtained were fitted to rheological models for non-Newtonian fluids, such as Ostwald de Waele, Bingham Plastic, and Herschel–Bulkley (Table 1).

### 2.7. Statical Analysis

The results were analyzed using an analysis of variance (ANOVA) with a 95% confidence level, and an LSD test was used to compare the treatments. Pearson’s correlation analysis was applied to determine the relationship between the physical and chemical properties. The PCA was performed to identify the main patterns of variation present in the properties. Origin Pro 2024b software (Origin Lab Corporation, Northampton, MA, USA) was used for graphical representation and statistical tests.

## 3. Results and Discussion

### 3.1. Physical–Chemical Properties

Chenopodium quinoa germination (Table 2) caused physical–chemical parameter variation (*p*-value < 0.05). A decrease in lightness, whiteness index, and increases in chromatic values a* and b* were observed. These changes are consistent with previous studies suggesting that pigment synthesis, non-enzymatic browning reactions, and release of cellular components during germination contribute to the observed color development [2,28,29,30,31].

The particle size distribution ranged from 30.72 to 91.46 µm for ungerminated samples and 46.10 to 77.50 µm for germinated samples, evidencing rupture of cellular integrity and reorganization of protein and starch structures [32,33,34,35]. The span ranged from 1.66 to 3.69 for the ungerminated sample and from 1.95 to 3.19 for the germinated sample, indicating a wide range of particle size distribution and a certain degree of dispersion [36].

The metabolic changes, generated during germination, increased the content of phenolic compounds (up to 30.81 mg GAE/100 g in RPQ) and flavonoids (108.53 mg QE/100 g in YAQ), activating biosynthesis pathways as a molecular defense mechanism against oxidative stress conditions [30,37,38,39,40,41]. This process increased the concentration of natural antioxidants and modified the macromolecular structure, significantly improving the bioavailability of the bioactive compounds [36,42,43,44,45].

Regarding the nutritional profile, germination caused structural and biochemical modifications that varied the composition of the grains. The germinated varieties BCQ (14.13%) and RPQ (12.79%) exhibited the highest protein content. At the structural level, enzymatic activation degraded storage macromolecules, initiating a molecular reorganization that modified nutrient bioavailability [3,46,47,48,49,50]. Depolymerization of starch by amylases converted polysaccharide molecules into simple sugars, reducing the carbohydrate content [18,51]. Likewise, fat decreased due to the mobilization of triacylglycerols and the breakdown of fatty complexes that support the metabolic processes of germination [18,51,52]. Ash content showed a slight increase, while water content decreased due to its contribution to metabolic processes [11]. These changes were accompanied by modifications in the mineral content, with an increase in the bioavailability of elements, such as iron (RPQ: 5.83 mg/100 g), potassium (RPQ: 524.00 mg/100 g), phosphorus (BCQ: 367.00 mg/100 g), calcium (LOQ: 93.70 mg/100 g), and magnesium (YMQ: 162.90 mg/100 g), as a consequence of the degradation of antinutritional compounds, such as phytic acid, responsible for mineral retention [51,53,54,55]. Finally, the reduction in water activity (Aw) after germination indicated a structural reorganization of the biopolymers [56], generating materials with greater microbiological stability [57].

### 3.2. Thermal Properties

**Figure 2:**
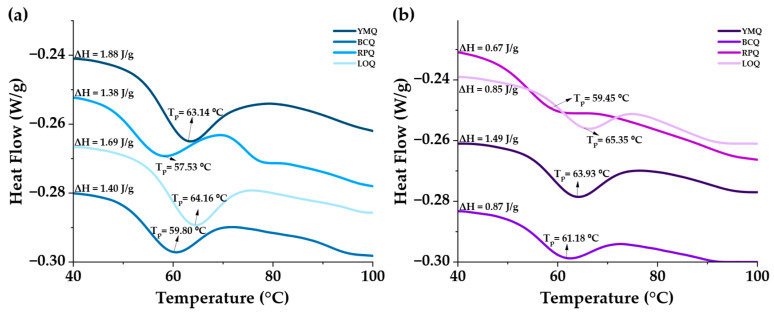
Temperature and enthalpy of gelatinization of quinoa: (**a**) ungerminated, (**b**) germinated.

**Figure 3:**
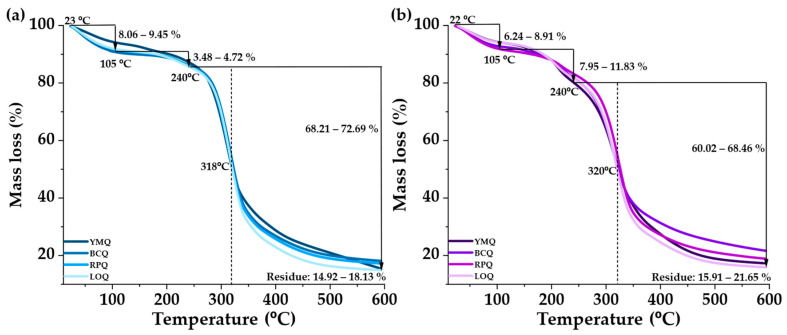
Thermal stability of quinoa: (**a**) ungerminated, (**b**) germinated.

Thermograms (Figure 2) showed differences in thermal behavior between ungerminated and germinated quinoa samples, reflected by a displacement of gelatinization temperature (Tp). This variation was from 57.53 °C to 59.45 °C for RPQ and from 64.16 °C to 65.35 °C for LOQ, related to the modifications caused by germination [18]. At the structural level, the activity of amylolytic enzymes partially degraded the starch granules, decreasing their crystallinity and altering their molecular organization. Theoretically, the energy needed to break hydrogen bonds should decrease, facilitating gelatinization at lower temperatures [17,18,58,59,60,61]. However, the endothermic curves (Figure 3b) displaced towards slightly higher temperatures due to the release of soluble compounds and the interaction between lipids and starch. In the presence of heat and moisture, amylose–lipid complexes are formed when lipids with their exposed fatty acid methyl ends are incorporated into the amylose helices. This generates a crystalline structure that requires more thermal energy to break down [62,63,64]. On the other hand, among the germinated samples, the YMQ variety (1.49 J/g) presented the highest enthalpy value (ΔH), indicating that it required greater thermal energy for starch gelatinization [65,66].

Thermogravimetric analysis (Figure 3) shows the thermal decomposition of germinated and ungerminated quinoa samples. The first stage corresponds to the evaporation of water, with weight losses at temperatures below 100 °C. At this point, differences were observed between the ungerminated samples (5.08–9.25%) and the germinated samples (6.24–8.91%) due to the structural reorganization of hydrophilic components, such as proteins and starch, which affect the water retention capacity [67]. In the second stage (up to 240 °C), lignocellulosic compounds such as cellulose and hemicellulose are degraded [68,69]. During this stage, germinated samples presented an increase in weight loss (7.98–11.83%) due to the higher concentration of peptides and simple sugars originating during germination. The compounds’ pyrolysis was triggered in the third stage, characterized by thermal degradation around 315–320 °C. The germinated samples showed a slight thermal displacement, indicative of the formation of more heat-resistant molecular structures, such as amylose–lipid complexes and protein cross-linking. This stage is the most extended stage, and mass loss slows down [70]. The final phase, close to 600 °C, shows an increase in the residue of the germinated samples (15.93–21.69%). This increase could be attributed to a higher mineral content, the formation of more stable structures, and modifications in the composition during germination.

### 3.3. Structural Properties

**Figure 4:**
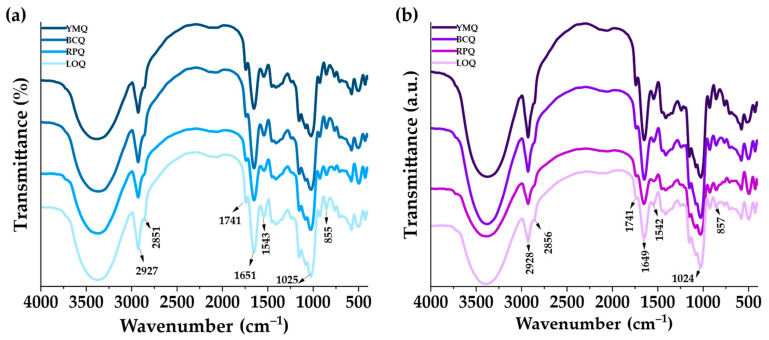
Functional groups of quinoa: (**a**) ungerminated, (**b**) germinated.

Vibrational analysis using infrared spectroscopy (Figure 4) showed subtle differences between quinoa varieties. In the ungerminated samples, variations were observed in the intensities of the bands associated with functional groups of starch (2927 cm^−1^, 2851 cm^−1^, 1025 cm^−1^, and 855 cm^−1^; –CH–, –CH_2_–, C–OH, and –OH, respectively) and proteins (1651 cm^−1^ and 1543 cm^−1^), reflecting differences in the biochemical composition and molecular organization of each variety [26,71,72,73,74]. During germination, a decrease in the intensity of the starch-related bands is observed, suggesting a partial depolymerization caused by enzymatic activity [56]. This phenomenon was especially noticeable in the BCQ and RPQ varieties, where reductions in starch crystallinity were more marked, indicating greater molecular reactivity. On the other hand, the bands corresponding to the amide groups I and II (1651 cm⁻^1^ and 1543 cm⁻^1^) showed a change in the YMQ and LOQ varieties, suggesting modifications in the secondary structure of the proteins and polymer induced by germination.

### 3.4. Rheological Properties

**Figure 5:**
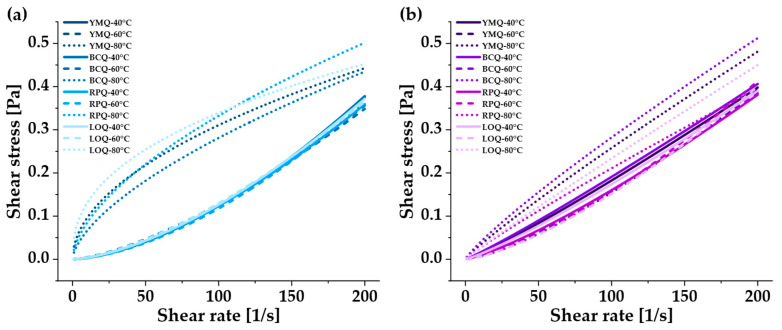
Flow properties: shear stress vs. shear rate of quinoa: (**a**) ungerminated, (**b**) germinated.

The rheological analysis (Figure 5) showed a non-Newtonian behavior of the quinoa samples, characterized by a non-linear relationship between shear stress and shear rate. At 40 °C and 60 °C, dilatant behavior (n > 1) was observed, suggesting molecular reorganization. This phenomenon occurs due to the dynamic alignment of the polymeric chains, where the interactions between chains intensify as the shear rate increases [75].

At 80 °C, the samples exhibited pseudoplastic behavior (n < 1), reflecting a partial dissociation of the semi-gelatinized starch structures and increased molecular mobility [76]. This flow type is especially useful in industrial processes, such as extrusion and molding, where controlled flow is needed to obtain uniform, high-quality products.

The rheological analysis (Table 3) showed a marked non-Newtonian character, which was satisfactorily adjusted to multiple rheological models: Ostwald de Waele (R^2^ < 0.93654), Bingham (R^2^ < 0.84859), and Herschel–Bulkley (R^2^ < 0.90059), with a particularly low sum of squared residuals (SSR) values for the Ostwald de Waele model.

A systematic decrease in η with increasing temperature was observed, reflecting an increasing pseudo-plasticity. This behavior shows molecular reorganization due to the reduction in flow resistance and increased molecular mobility at elevated temperatures, facilitating decomposition and structural alignment under shear [54,76].

### 3.5. Pearson Correlation

**Figure 6:**
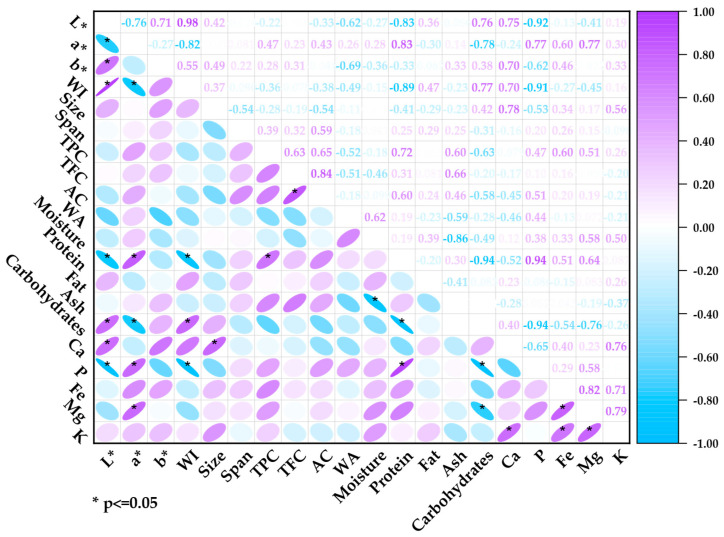
Pearson correlation.

Pearson’s correlation analysis (Figure 6) showed relationships between the properties of quinoa. Variables such as lightness (L*) and whiteness index (WI) exhibited a significant positive correlation, reflecting the structural clarity of quinoa; on the other hand, the chromatic components a* and b* had negative correlations with L* and WI, indicating that higher color saturation is associated with a decrease in lightness.

Regarding the metabolic and nutritional aspects, a negative correlation was observed between proteins and carbohydrates and a positive correlation between minerals (Ca, P, Fe, Mg, and K), evidencing a mechanism of compensation and metabolic regulation during germination. The bioactive compounds presented moderate positive correlations with the protein content, indicating a relationship with protein metabolism, the synthesis of phenolic compounds, and metabolic transformations inherent to germination. Also, a strong positive correlation was shown between total flavonoids and antioxidant capacity and negative correlations between optical properties and bioactive compounds, suggesting that a higher content of these compounds darkens the samples [40].

Hydration parameters such as humidity and water activity showed positive and negative correlations with other parameters, suggesting that water content influences molecular transformations and regulates metabolic processes.

### 3.6. Principal Component Analysis

**Figure 7:**
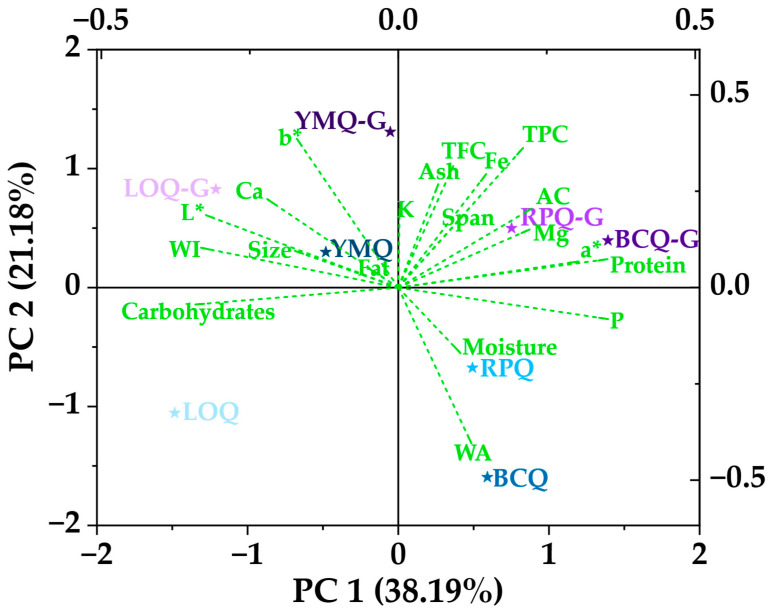
Principal component analysis of quinoa.

The principal component analysis (PCA) presented in Figure 7 provided a broader view of quinoa varieties’ physicochemical, nutritional, and functional properties, including germinated varieties. The first principal component (PC1), which explains 38.19% of the variance, is mainly associated with nutritional and functional properties. In this component, parameters such as proteins, phenolic compounds (TPC), flavonoids (TFC), and antioxidant capacity (AC) are at the positive end, while the variables of lightness (L*) and whiteness index (WI) predominate at the opposing end. This suggests that a higher content of bioactive compounds is related to lower luminosity and whiteness [29,40].

The second principal component (PC2), which explains 21.18% of the variance, is mainly related to physical and mineral characteristics. In the upper part of the graph, Ca, Fe, K, and particle size are grouped, while water activity (WA) and moisture content predominate in the lower part.

Germinated varieties tend to concentrate in the upper right quadrant, indicating increased bioactive compounds and antioxidant capacity after germination [36,42,45]. In particular, YMQ-G showed the highest differentiation in PC2, reflecting possible changes in mineral content and physical characteristics after germination. On the other hand, BCQ and RPQ were located in the lower right quadrant, associated with higher humidity and water activity. The strong correlation between TPC, TFC, and AC suggests that phenolic compounds and flavonoids mainly contribute to quinoa’s antioxidant capacity [21,77].

## 4. Conclusions

Germination significantly modified the physical–chemical, thermal, structural, and rheological properties of the four varieties of Andean quinoa. The results revealed increases in protein bioavailability, phenolic compounds, and antioxidant capacity, along with molecular reorganization reflected in rheological and thermal behavior. These modifications are attributable to the formation of amylose–lipid complexes, reduced starch crystallinity, and increased soluble compounds, improving germinated quinoa’s functionality for functional food and biomaterial applications. Future research could focus on optimizing germination conditions, detailed study of molecular interactions that contribute to improving their properties, and evaluating their performance on an industrial scale.

## Figures and Tables

**Table 1:** Rheological models for non-Newtonian fluids.

Model	Equation
Ostwald de Wale	$\tau =k\gamma^{n}$
Bingham Plastic	$\tau =\tau_{y}+\eta_{B}\gamma$
Herschel–Bulkley	$\tau =\tau_{y}+k_{H}\gamma^{n}$

τ is shear stress, γ is shear rate, n is Flow behavior index, k is consistency index, $\tau_{y}$ is the elastic limit, $\eta_{B}$ is plastic viscosity, and $k_{H}$ is consistency index.

**Table 2:** Physical–chemical properties.

Variety	YMQ			BCQ			RPQ			LOQ		
Property	$\bar{x}$	±	s	$\bar{x}$	±	s	$\bar{x}$	±	s	$\bar{x}$	±	s
Ungerminated												
L*	83.47	±	0.02 ^a^	70.10	±	0.05 ^b^	71.74	±	0.05 ^c^	82.99	±	0.02 ^d^
a*	0.88	±	0.00 ^a^	1.75	±	0.01 ^b^	3.69	±	0.01 ^c^	0.80	±	0.01 ^d^
b*	16.61	±	0.02 ^a^	9.41	±	0.02 ^b^	12.46	±	0.01 ^c^	16.34	±	0.02 ^d^
WI	76.55	±	0.02 ^a^	68.61	±	0.05 ^b^	68.90	±	0.05 ^c^	76.40	±	0.03 ^d^
Size (µm)	43.16	±	1.80 ^a^	30.72	±	0.47 ^b^	82.74	±	2.45 ^c^	91.46	±	0.81 ^d^
Span	3.39	±	0.14 ^a^	1.76	±	0.02 ^b^	1.99	±	0.03 ^c^	1.66	±	0.01 ^d^
TPC (GAE/100 g db)	25.35	±	0.62 ^a^	23.70	±	0.89 ^b^	22.20	±	0.26 ^c^	15.72	±	0.58 ^d^
TFC (mg QE/100 g db)	59.82	±	0.63 ^a^	61.59	±	0.63 ^b^	44.62	±	2.54 ^c^	51.20	±	1.09 ^d^
AC (µmol TE/g db)	146.49	±	1.87 ^a^	141.18	±	2.33 ^b^	86.90	±	1.84 ^c^	42.15	±	0.96 ^d^
WA	0.37	±	0.002 ^a^	0.46	±	0.001 ^b^	0.45	±	0.001 ^c^	0.47	±	0.001 ^d^
Moisture (%)	8.76	±	0.11 ^a^	8.13	±	0.08 ^b^	9.04	±	0.03 ^c^	8.37	±	0.10 ^d^
Protein (%)	10.53	±	0.03 ^a^	12.53	±	0.08 ^b^	12.19	±	0.05 ^c^	8.64	±	0.04 ^d^
Fat (%)	6.46	±	0.01 ^a^	5.92	±	0.04 ^b^	5.81	±	0.01 ^c^	5.63	±	0.04 ^d^
Ash (%)	2.05	±	0.03 ^a^	2.22	±	0.03 ^b^	1.96	±	0.02 ^c^	1.91	±	0.02 ^d^
Carbohydrates (%)	72.20	±	0.10 ^a^	71.20	±	0.14 ^b^	71.00	±	0.00 ^c^	75.45	±	0.03 ^d^
Ca (mg/100 g)	90.80	±	0.20 ^a^	53.00	±	1.00 ^b^	89.00	±	1.00 ^c^	92.10	±	0.10 ^d^
P (mg/100 g)	249.70	±	0.36 ^a^	361.00	±	1.00 ^b^	328.00	±	0.44 ^c^	170.20	±	0.10 ^d^
Fe (mg/100 g)	4.95	±	0.03 ^a^	4.18	±	0.07 ^a^	5.15	±	0.14 ^a^	4.67	±	0.11 ^a^
Mg (mg/100 g)	154.50	±	1.80 ^a^	132.50	±	0.50 ^a^	190.50	±	0.50 ^a^	121.60	±	0.66 ^a^
K (mg/100 g)	471.60	±	1.23 ^a^	391.10	±	0.85 ^a^	510.90	±	1.01 ^a^	431.80	±	0.26 ^a^
Germinated												
L*	81.64	±	0.00 ^a^	69.07	±	0.02 ^b^	74.20	±	0.01 ^c^	84.21	±	0.01 ^d^
a*	2.50	±	0.01 ^a^	3.54	±	0.02 ^b^	2.94	±	0.01 ^c^	1.09	±	0.01 ^d^
b*	17.16	±	0.02 ^a^	15.49	±	0.02 ^b^	16.80	±	0.02 ^c^	18.73	±	0.06 ^d^
WI	74.74	±	0.01 ^a^	65.23	±	0.02 ^b^	69.07	±	0.02 ^c^	75.48	±	0.05 ^d^
Size (µm)	75.56	±	2.41 ^a^	46.10	±	1.39 ^b^	75.64	±	1.61 ^c^	77.50	±	1.58 ^d^
Span	2.11	±	0.08 ^a^	3.19	±	0.08 ^b^	1.99	±	0.03 ^c^	1.95	±	0.02 ^d^
TPC (GAE/100 g db)	29.83	±	0.39 ^a^	29.58	±	0.92 ^b^	30.81	±	0.78 ^c^	26.40	±	0.96 ^d^
TFC (mg QE/100 g db)	108.53	±	2.17 ^a^	94.42	±	2.25 ^b^	57.37	±	1.27 ^c^	68.67	±	1.63 ^d^
AC (µmol TE/g db)	225.00	±	2.06 ^a^	241.43	±	2.32 ^b^	101.19	±	1.65 ^c^	68.00	±	2.92 ^d^
Water activity	0.32	±	0.001 ^a^	0.42	±	0.001 ^b^	0.41	±	0.001 ^c^	0.28	±	0.002 ^d^
Moisture (%)	7.97	±	0.05 ^a^	7.62	±	0.04 ^b^	8.93	±	0.03 ^c^	6.46	±	0.04 ^d^
Protein (%)	11.81	±	0.03 ^a^	14.13	±	0.06 ^b^	13.75	±	0.14 ^c^	10.06	±	0.05 ^d^
Fat (%)	6.22	±	0.02 ^a^	5.40	±	0.01 ^b^	5.64	±	0.07 ^c^	5.53	±	0.01 ^d^
Ash (%)	2.36	±	0.04 ^a^	2.69	±	0.01 ^b^	2.15	±	0.04 ^c^	2.72	±	0.01 ^d^
Carbohydrates (%)	71.64	±	0.01 ^a^	70.16	±	0.07 ^b^	69.53	±	0.12 ^c^	75.24	±	0.07 ^d^
Ca (mg/100 g)	93.70	±	0.61 ^a^	60.00	±	1.00 ^b^	92.10	±	1.01 ^c^	93.70	±	0.70 ^d^
P (mg/100 g)	262.80	±	0.17 ^a^	367.00	±	1.73 ^b^	346.50	±	1.32 ^c^	178.40	±	0.61 ^d^
Fe (mg/100 g)	5.15	±	0.04 ^a^	5.27	±	0.05 ^b^	5.83	±	0.07 ^c^	4.76	±	0.08 ^d^
Mg (mg/100 g)	162.90	±	0.17 ^a^	157.20	±	0.98 ^b^	192.87	±	0.81 ^c^	131.50	±	0.50 ^d^
K (mg/100 g)	486.80	±	0.26 ^a^	402.40	±	0.60 ^b^	524.00	±	1.00 ^c^	447.80	±	0.35 ^d^

$\bar{x}$ is mean, s is standard deviation, YMQ is Quinoa Yellow Marangani, BCQ is Quinoa Black Collana, RPQ is Quinoa Red Pasankalla, and LOQ is Quinoa Lord of Orchard. Equal letters mean that there is no significant difference, evaluated through the Tukey test, with α = 5%.

**Table 3:** Rheological parameters for non-Newtonian fluids and statics.

Model	Parameter	40 °C	60 °C	80 °C	40 °C	60 °C	80 °C	40 °C	60 °C	80 °C	40 °C	60 °C	80 °C
		YMQ			BCQ			RPQ			LOQ		
		Ungerminated											
Ostwald de Waele	k (Pa·s *^n^*)	0.0001	0.0001	0.0295	0.0001	0.0002	0.0155	0.0001	0.0001	0.0216	0.0001	0.0001	0.0500
	η	1.5856	1.4690	0.5110	1.6119	1.4465	0.6289	1.5396	1.6260	0.5936	1.5606	1.4900	0.4158
	$R^{2}$	0.9983	0.9860	0.9621	0.9853	0.9853	0.9652	0.9821	0.9863	0.9618	0.9837	0.9865	0.9365
	SSR	0.0043	0.0321	0.0789	0.0377	0.0322	0.0829	0.0421	0.0326	0.1132	0.0408	0.0325	0.1170
Bingham	$\tau_{y}$ (Pa)	0.0000	0.0000	0.1198	0.0000	0.0000	0.0818	0.0000	0.0000	0.1079	0.0000	0.0000	0.1578
	$\eta_{B}$ (Pa·s *^n^*)	0.0018	0.0018	0.0018	0.0018	0.0017	0.0019	0.0018	0.0018	0.0021	0.0018	0.0018	0.0016
	$R^{2}$	0.8658	0.8855	0.9752	0.8486	0.8913	0.9690	0.8627	0.8415	0.9813	0.8584	0.8800	0.9572
	SSR	0.3349	0.2630	0.0517	0.8494	0.2387	0.0739	0.3219	0.3771	0.0554	0.3549	0.2882	0.0790
Herschel–Bulkley	$\tau_{y}$ (Pa)	0.0000	0.0000	0.0913	0.0000	0.0000	0.0548	0.0000	0.0000	0.0965	0.0000	0.0000	0.1268
	$k_{H}$ (Pa·s *^n^*)	0.0006	0.0017	0.0051	0.0016	0.0018	0.0049	0.0022	0.0016	0.0032	0.0014	0.0017	0.0055
	H	1.2121	0.9798	0.8059	1.0049	0.9703	0.8244	0.9328	0.9897	0.9256	1.0197	0.9893	0.7804
	$R^{2}$	0.9725	0.9280	0.9799	0.9113	0.9286	0.9732	0.9006	0.9045	0.9819	0.9234	0.9275	0.9625
	SSR	0.0683	0.1646	0.0416	0.2253	0.1561	0.0636	0.2318	0.2262	0.0534	0.1911	0.1734	0.0688
		Germinated											
Ostwald de Waele	k (Pa·s *^n^*)	0.0010	0.0003	0.0041	0.0013	0.0004	0.0054	0.0005	0.0002	0.0032	0.0008	0.0002	0.0030
	H	1.1250	1.3442	0.8999	1.0890	1.2933	0.8603	1.2515	1.4009	0.9104	1.1655	1.3986	0.9455
	$R^{2}$	0.9860	0.9826	0.9836	0.9814	0.9840	0.9725	0.9685	0.9852	0.9788	0.9682	0.9797	0.9846
	SSR	0.0359	0.9827	0.0563	0.0498	0.0401	0.1039	0.0787	0.0430	0.0504	0.0801	0.0543	0.0481
Bingham	$\tau_{y}$ (Pa)	0.0000	0.0000	0.0300	0.0000	0.0000	0.0421	0.0000	0.0000	0.0218	0.0000	0.0000	0.0179
	$\eta_{B}$ (Pa·s *^n^*)	0.0019	0.0019	0.0023	0.0020	0.0019	0.0024	0.0019	0.0020	0.0019	0.0019	0.0019	0.0022
	$R^{2}$	0.9804	0.9348	0.9913	0.9784	0.9458	0.9849	0.9401	0.9212	0.9846	0.9592	0.9216	0.9883
	SSR	0.0502	0.1686	0.0297	0.0577	0.1358	0.0572	0.1495	0.2278	0.0366	0.1029	0.2099	0.0367
Herschel–Bulkley	$\tau_{y}$ (Pa)	0.0322	0.0261	0.0558	0.0000	0.0221	0.0724	0.0000	0.0237	0.0407	0.0329	0.0300	0.0392
	$k_{H}$ (Pa·s *^n^*)	0.0002	0.0001	0.0008	0.0022	0.0001	0.0007	0.0012	0.0001	0.0007	0.0002	0.0000	0.0009
	H	1.3937	1.6123	1.2038	0.9781	1.5046	1.2336	1.0834	1.6354	1.1754	1.4691	1.7291	1.1715
	$R^{2}$	0.9930	0.9889	0.9952	0.9764	0.9883	0.9898	0.9604	0.9901	0.9874	0.9759	0.9883	0.9910
	SSR	0.0178	0.0286	0.0164	0.0626	0.0292	0.0383	0.0984	0.0284	0.0299	0.0606	0.0311	0.0279

n is flow behavior index, k is consistency index, $\tau_{y}$ is the elastic limit, $\eta_{B}$ is plastic viscosity, $k_{H}$ is consistency index, and SSR is sum of squared residuals.

## Data Availability

The data are available in the article; further queries can be directed to the corresponding author.
